# Laser resonance frequency analysis of pedicle screw stability: A cadaveric model bone study

**DOI:** 10.1002/jor.24983

**Published:** 2021-01-28

**Authors:** Daisuke Nakashima, Katsuhiro Mikami, Shunsuke Kikuchi, Masaharu Nishikino, Toshiyuki Kitamura, Noboru Hasegawa, Morio Matsumoto, Masaya Nakamura, Takeo Nagura

**Affiliations:** ^1^ Department of Orthopedic Surgery Keio University School of Medicine, Shinjuku Tokyo Japan; ^2^ Faculty of Biology‐Oriented Science and Technology, Kindai University Kinokawa Wakayama Japan; ^3^ The National Institutes for Quantum and Radiological Science and Technology Quantum Beam Science Research Directorate Kansai Photon Science Institute Kyoto Japan; ^4^ Department of Clinical Biomechanics Keio University School of Medicine, Shinjuku Tokyo Japan

**Keywords:** fixation force, implant failure, laser, loosening, multiple operated back, resonance frequency analysis

## Abstract

There is no evaluation method currently available to assess intraoperative pedicle screw fixation (PSF) strength. In this study, we established a laser‐based resonance frequency analysis (RFA) system with high‐speed, noncontact, quantitative measurements of PSF. Clinical investigations in the future can assess surgical failure risk of implants. We investigated the characteristics of the laser RFA and compared them with the conventional methods. We inserted a pedicle screw in the vertebral pedicle of human cadaver or model bone, followed by screw pull‐out, peak torque, implant stability quotient (ISQ) value obtained by the magnetic dental RFA system, and fixation force of laser RFA. We compared the outcomes using best‐fit linear or logarithmic approximations. For the model bone study, the resonance frequency (RF) versus peak torque/pull‐out force (POF) demonstrated strong correlations using logarithmic approximation (vs. peak torque: *R* = 0.931, *p* < .001, vs. POF: *R* = 0.931, *p* < .001). RF strongly correlated with the ISQ value using linear approximation (*R* = 0.981, *p* < .001). For the cadaveric vertebrae study, the correlation coefficients between RF and the peak torque/POF were significant regardless of approximation method (peak torque: logarithmic: *R* = 0.716 vs. linear: *R* = 0.811; *p* < .001) (POF: logarithmic: *R* = 0.644 vs. linear: *R* = 0.548; *p* < .05). Thus, the results of this study revealed a constant correlation between RFA and conventional methods as a measurement validation, predicting favorable support for intraoperative PSF. RFA has the potential to be a new index for evaluating the implant fixation force.

## 1 INTRODUCTION

The stability of an implant is an essential factor in orthopedic implant surgery as it ensures anchorage to the bone.^1^, ^2^ With the aging population, there has been an increase in the annual number of spinal surgeries.^3^, ^4^ However, one of the detrimental factors in spinal instrumentation surgery is pedicle screw loosening, and the prevalence of implant failure is as high as ~12%.^5^ The rate of revision spinal surgery has been reported to be 40%,^6^ and implant failure is one of the contributing factors. To address these limitations, we should evaluate the initial fixation of the implant and assess the risk factors for loosening. To date, there have been no intraoperative methods to quantitatively assess the initial stability of the pedicle screw on the vertebra.

Pull‐out force (POF)^7^ and insertion torque^1^, ^8^ are the tests to assess fixation forces and evaluate the implant stability. POF is a parameter that reflects the screw fixation strength and is measured destructively via mechanical testing in the laboratory. POF is invasive and cannot be measured intraoperatively. On the contrary, insertion torque can be measured during operation; however, more than one measurement after the screw has been fixed to the bone cannot be performed. As the surgeon and the assistant routinely double‐check with a sounder after the tap; therefore, it is important to establish a system that enables multiple checks. Hence, POF and insertion torque cannot be used for performing intraoperative measurements.

Attempts have been made to develop an intraoperative implant stability evaluation method, including studies to analyze the resonance frequency (RF) by vibrating the implant with some external force, followed by RF measurement. This method is referred to as resonance frequency analysis (RFA). The most notable assessment, which is used in the engineering field, is the percussion method wherein the sound of a tunnel wall is determined by the sound of hitting the wall with a hammer.

To date, the medical application of RFA has only been with dental implants.^9^, ^10^ Osstell ISQ® (Osstell, Integration Diagnostics) has been used as an RFA for dental implants by vibrating the implant with a magnetic pulse.^10^, ^11^, ^12^, ^13^, ^14^ Subsequently, the results are displayed as a proprietary parameter, with the implant stability quotient (ISQ) ranging from 0 (lowest level of stability) to 100 (highest level of stability).^14^ The RF is about 3000 Hz when the ISQ value is 0 and about 8000 Hz when the ISQ value is 100.^15^ This noninvasive and highly reproducible technique can reflect a multidirectional fixation force.^14^, ^16^, ^17^

There are multiple studies in which noninvasive measurements of orthopedic implant stability in orthopedic surgery have been performed in the laboratory.^18^, ^19^, ^20^, ^21^, ^22^, ^23^, ^24^, ^25^ A vibration‐based method was proposed by Lippmann^26^ in 1932 and has been studied by several researchers. The vibration analysis of the femoral stem and acetabular cup during total hip replacement has been studied with regard to hip arthroplasty.^20^, ^21^, ^22^, ^24^ For knee arthroplasty, Leuridan et al.^20^ reported the potential of the vibration‐based method to evaluate knee prosthesis stability.^27^ Subcutaneous accelerometers can be used postoperatively to detect vibrations from a device positioned on the skin.^20^ Despite several experiments, there have not been methods that could be used in orthopedic clinical practice. There are several limitations of the previous methods, that is, they require massive vibrating units, including vibrator and acceleration sensors, and the vibration methods can only be used to determine stability.^20^, ^22^, ^28^ The unwieldy and complicated nature of this approach renders its clinical use as quantitative methods impossible.

The magnetic RFA (the ISQ value) already in use in dentistry requires a smart peg to be attached before measurement, thus making it difficult to use deep within the body, other than in the oral cavity where it can easily access the implant.^29^, ^30^, ^31^ We developed an RFA system using a pulse laser and laser Doppler vibrometer, which is completely contactless and does not involve the attachment of devices, such as magnets, directly to the implant. Laser RFA is used to inspect internal defects in concrete structures, such as tunnels. It has been used as a laser remote‐sensing approach.^32^, ^33^

The basic principle involves the irradiation of an evaluation sample with a pulse laser to induce vibration on the surface through the laser ablation process and the subsequent detection of the induced vibration using a laser Doppler vibrometer. The scheme of laser RFA is similar to that of a hammering inspection, and it can provide high‐speed, noncontact, and quantitative measurements. The advantages of an impact scheme using laser pulse irradiation have been previously reported and shown to be massless and remote laser sensing is comparable to physical excitation measurements.^34^

In this study, we sought to investigate the characteristics of laser RFA using biomechanical test materials (artificial bone) and human cadaveric vertebrae and compare the technique with mechanical test forces (insertion torque and POF) and the ISQ value.

## 2 MATERIALS AND METHODS

### 2.1 Biomechanical test materials

Five types of solid rigid polyurethane forms and one type of cellular polyurethane form (biomechanical test materials) (SAWBONES, Pacific Research Laboratory, Inc.) were prepared as test materials to represent diverse human vertebrae (Table 1). Five test materials of each type were prepared for a total of 30.

**Table 1:** Biomechanical test materials

Catalog no.	Density (lb/ft^3^)	Sensitivity (g/cm^3^)
Solid rigid polyurethane forms (five blocks per type prepared)		
#1522‐23	5	0.08
#1522‐01	10	0.16
#1522‐48	12	0.192
#1522‐03	20	0.32
#1522‐03	30	0.48
Cellular polyurethane forms (prepared five blocks)		
#1522‐10	10	0.16

These biomechanical test materials were homogeneous and consistent with human cancellous bone.^35^ They were stacked 60 mm × 60 mm × 40 mm and conformed to the American Society for Testing and Materials (ASTM) standard.

### 2.2 Human cadaveric vertebrae

**Figure 1:**
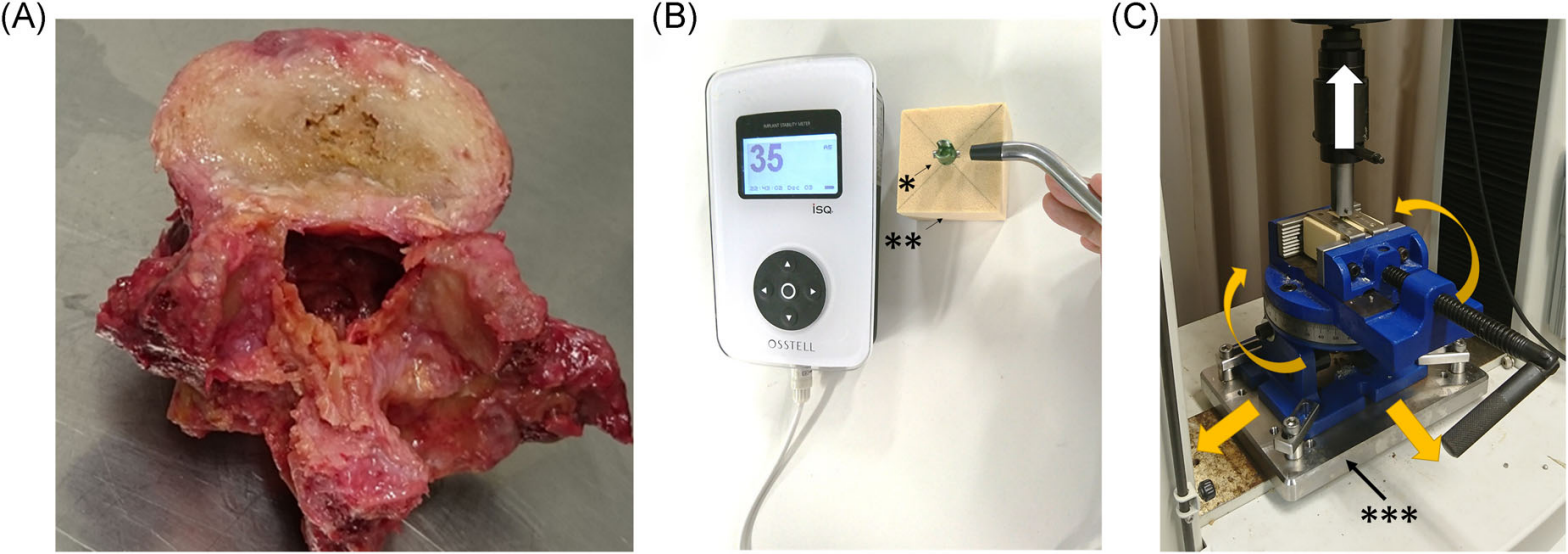
Materials and test forces for pedicle screw fixation. A, Human cadaveric vertebra. B, The implant stability quotient value measured by the Osstell apparatus. C, Pull‐out force set up: The material is pulled with a special fixture without being pushed from the side. The base plate moves freely and combines the vise at a freely determined angle (yellow allows); instead of directly sandwiching the material in a vise, it is structured to support the top surface of the material with a plate. Pedicle screw, **biomechanical test material, ***base plate [Color figure can be viewed at wileyonlinelibrary.com]

For the experimental use of fresh, nonfrozen human cadavers, written informed consent was obtained from each donor as per the ethical guidelines of Keio University School of Medicine. The cadavers were stored at <4°C shortly after death and dissected within seven days of death. The discs and ligaments were removed from the spine to obtain the vertebrae (Figure 1A). All experiments were approved by our institution's ethics committee (approval number: 20150385). Two human lumbar spinal sections (L1–L5, 10 vertebrae) were used in this study (donor age, 89 and 76; one male and one female). The intervertebral discs and ligaments were dissected from the vertebrae. One vertebra that was damaged during preparation was excluded. The remaining vertebrae were visually inspected and diagnosed by a spinal surgeon (13 years of experience), and there were no vertebrae showing fractures or spinal metastases. We used nine vertebrae and two pedicle screws were placed per vertebrae for a total of 18.

### 2.3 Pedicle screw insertion

We prepared single‐threaded, non‐cannulated, titanium alloy (Ti‐6Al‐4V [ELI], ASTM F136), monoaxial pedicle screws (Catalog no. CMS05135; Kyocera Medical Corporation) with lengths of 45 mm, an outer threaded diameter of Ø 5.5 mm, an inner thread diameter start point of Ø 3.8 mm (an endpoint of Ø 4.6 mm), and a screw pitch of 2.5 mm. Using an awl, we made a small hole in the target area where a model bone or vertebra screw was to be inserted at the center of the 60 mm × 60 mm material and the entry point of the vertebral pedicle.

**Figure 2:**
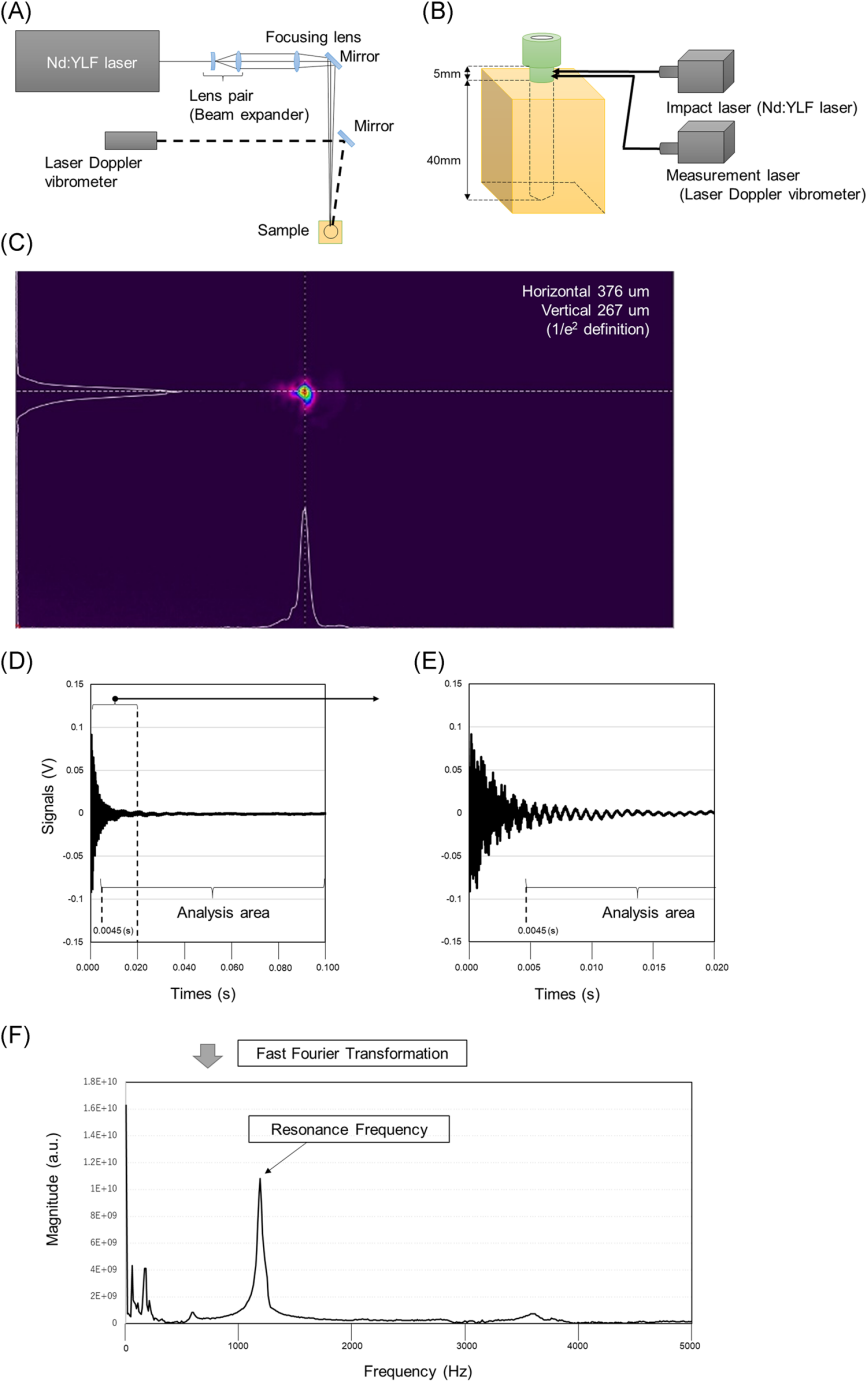
Schematic representation of the set up used for laser resonance frequency analysis for pedicle screw stability, raw waveform data, and the analyses of resonance frequency. A, The layout of the laser system. B, Pedicle screw insertion and irradiation scheme of both laser systems to pedicle screw samples. The screw is inserted 40 mm and the root of the screw part is left 5 mm. C, The laser system with a pulse laser and a laser Doppler vibrometer. The beam profile of the Nd:YLF laser at a focus point that is, irradiation pattern for the pedicle screw. D, Waveform raw data. E, Data ranging from 0.0045 s to 0.1 s after laser irradiation were purged to obtain a clear signal for the analysis. F, Frequency spectra of laser‐induced vibration of the pedicle screw. The resonance frequency is defined as the frequency of the highest magnitude in the range of 1000–5000 Hz [Color figure can be viewed at wileyonlinelibrary.com]

Before insertion, a 1.5 mm drill bit was used to drill a pilot hole in the material. A pedicle screw was inserted at its 40‐mm length, and the root of the screw was left 5 mm (Figure 2B). Paik et al.^36^ reported that if the screw head is inserted until it comes into contact with bone, the screw head causes bone destruction and reduces the fixation force. Therefore, the technique used in our study did not allow the screw head to come into contact with the specimen, similar to clinical situations. All screws were inserted at the same depth (40 mm) using a consistent depth gauge. The detailed implant stability measurements (POF and insertion torque) procedures were as previously described.^31^

### 2.4 Insertion torque measurement

A digital torque gage HTGA‐5N (IMADA Co., Ltd.) was used to measure the insertion torque (peak torque)^31^, ^37^, ^38^ at the 40‐mm insertion. The specifications of this torque gage were as follows: accuracy ± 0.5% full‐scale ± 1 digit and sampling rate 2000 data/s. We measured the insertion torque (Nm) while advancing the screw into the material, was it was reported to progressively increase with an increase in the number of penetrating screw threads as the screw advanced. Thus, maximum torque was achieved when the screw was inserted to the appropriate length. The surgeon felt the maximum torque to be the strength of the screw fixation, defined as the peak torque.^31^, ^37^, ^39^

### 2.5 Resonance frequency analysis with an osstell apparatus

Magnetic RFA was performed using a specific device (Osstell ISQ, Osstell) after the pedicle screw insertion was complete and without contacting the screw, per previous reports.^31^ During measurements, the material was placed on a standard laboratory table rather than being held in a fixture (Figure 1B). We did not use a fixture in this study because the pressure of the fixture could change the ISQ value. The ISQ value was obtained from the Osstell ISQ®. The range was from 0 to 100 depending on the RF (Hz) of the pedicle screws. The higher the ISQ value, the more stable the implant.^40^ The pedicle screws were vibrated via a micro‐electromagnetic wave, which generated inertial forces due to the mass of the magnets in a plane radial to the axis of the screw. The size of the magnet was scaled according to the size of the pedicle screw relative to the dental implant.^31^

### 2.6 Laser resonance frequency analysis

The experimental layout of the laser system is shown in Figure 2A,B. The laser spot size of the impact laser pulse was adjusted by a beam expander. The adjusted beam was then focused with a focusing lens. The beam diameters of the focusing spot were 0.65 mm (horizontal) and 0.50 mm (vertical). These values were defined as the 1/*e* ^2^ values from the peak values of the Gaussian fitting for the bean profile (Figure 2C). The wavelength and pulse width of the impact laser was 1053 nm and 10 ns (full width at half maximum), respectively. The irradiation pulse energy was set to 46 mJ and was evaluated with an energy meter (QE25LP‐H‐MB‐QED, Gentec Electro‐Optics, Inc.).

The laser used in this study had less than 1/1000th of the average power compared to incisional devices, such as the laser scalpel. However, because it is a class 3B laser device, according to International Electrotechnical Commission standards, laser protection glasses were required for the surgeon. The pulse laser system was operated at a repetition rate of 10 Hz. The laser Doppler vibrometer was continuously irradiated to detect the induced vibration. Both lasers were irradiated on the neck of the pedicle screw sample (Figure 2B). The irradiation points between the pulse laser and laser Doppler vibrometer had a displacement of 1 mm to inhibit external noises, such as a laser ablation plume and plasma emission. The signal measurement from the laser Doppler vibrometer was captured with a multi‐function measuring system (RioNote, RION Co., Ltd.). It was synchronized with the timing of laser pulse irradiation (Q‐switch operation signal), and it was saved for 1.6 s, that is, data of 16 pulse irradiations. The signal obtained included laser‐induced vibrations with 16 laser pulses, divided into individual pulse irradiation and averaged with all divided data. Data acquired until 4.5 ms after laser irradiation among the analysis data were purged to obtain a clear signal of the fundamental and low‐order coefficient of variations (CVs) in the sound area. With a short period of impact, a laser pulse width (10 ns) has the potential to induce a wide spectrum of vibrations until ultrasonic vibration (~ MHz) (Figure 2D,E). Finally, the purged analysis data were analyzed by fast Fourier transform to obtain a frequency spectrum with a rectangular window function (Figure 2F).

### 2.7 Pull‐out force measurement

POF measurements were conducted as per the ASTM‐F543‐07 testing standards.^39^ The materials were set on a specially designed fixture with a self‐aligning function to keep a vertical pull‐out alignment (Figure 1C). The AG‐IS 10kN (Shimadzu Corporation) was used to measure the maximum POF at a testing speed of 5 mm/min.^39^

### 2.8 Statistical data analysis

The correlation analyses among the four fixation‐force measurements (peak torque, ISQ value, RF, and POF) were performed. We described two types of best‐fit approximations: linear and logarithmic. Correlation coefficient values < 0.3, 0.3–0.7, and >0.7 were defined as weak, moderate, and strong relationship, respectively. Statistical analyses were performed using SPSS statistics software version 26 (International Business Machines Corporation). The significance level of all tests was set at *p* ≤ .05.

## 3 RESULTS

The primary purpose of this study was to investigate the characteristics of Laser RFA; therefore, results not obtained with the Laser RFA are described in Figure S1.

**Figure 3:**
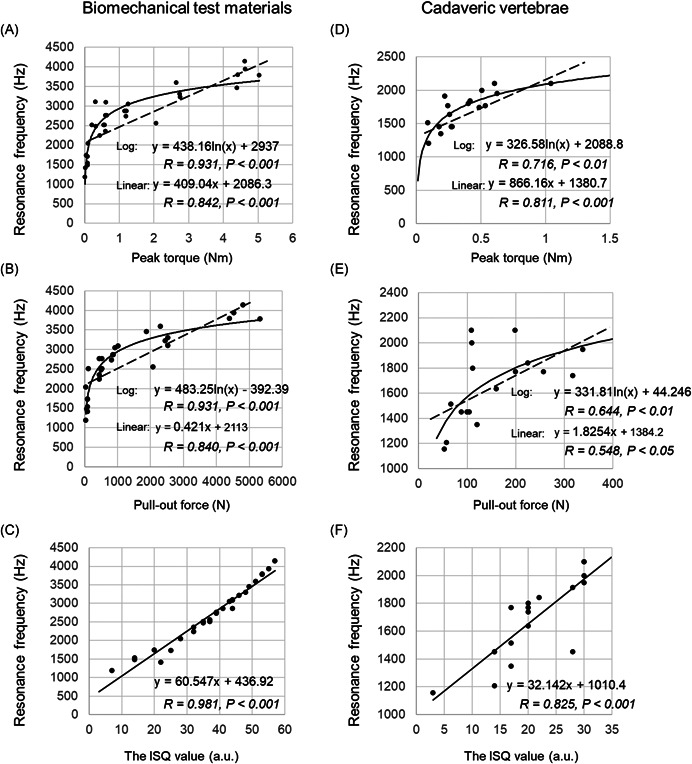
Linear and logarithmic correlation analyses among the four fixation force measures (resonance frequency, peak torque, pull‐out force, and the implant stability quotient value). A and D, Resonance frequency (Hz) versus peak torque (Nm). B and E, Resonance frequency (Hz) versus pull‐out force (N). C and F, resonance frequency versus implant stability quotient (ISQ) value (a.u.). The left‐hand column indicates the study using biomechanical test materials. The right‐hand column indicates the study using cadaveric vertebrae. a.u., arbitrary units; N, Newton, Nm, Newton meter

We used linear and logarithmic analyses to compare RF and conventional methods (peak torque, POF, and the ISQ value) (Figure 3). The RF versus peak torque and POF showed strong positive correlations regardless of the type of approximation. However, the logarithmic approximation showed stronger correlations (peak torque: *R* = 0.931, *p* < .001; POF: *R* = 931, *p* < .001) compared to the linear approximation (peak torque: *R* = 0.842, *p* < .001; POF: *R* = 0.840, *p* < .001) (Figure 3A,B). Furthermore, only a linear approximation was performed for RF versus the ISQ value (Figure 3C). The result showed that the RF strongly correlated with the ISQ value (*R* = 0.981, *p* < .001).

In the comparison between RF and peak torque in the cadaveric vertebrae study, the linear approximation was more strongly correlated than the logarithmic approximation (linear: *R* = 811, *p* < .001; logarithmic: *R* = 0.716, *p* < .01) (Figure 3D). Nevertheless, the comparison between RF and POF showed that the correlation coefficient was stronger for the logarithmic approximation compared to the linear approximation (logarithmic: *R* = 0.644, *p* < .01; linear: *R* = 548, *p* < .05) (Figure 3E). Finally, the correlation analysis between the RF and the ISQ value showed a strong correlation (*R* = 0.825, *p* < .001) (Figure 3F). RF and the ISQ values showed similar behavior in both, model bones and cadaveric vertebrae. Table 2 summarizes the comparison of the correlation coefficients between the four test forces.

**Table 2:** Correlation coefficients of resonance frequency and the ISQ value with respect to other test forces

	The ISQ value	Peak torque	Pull‐out force
Resonance frequency	0.981 (linear)**	0.931 (log)**	0.931 (log)**
The ISQ value	–	0.921 (log)**	0.777 (linear)**
Peak torque	–	–	0.920 (linear)**
pull‐out force	–	–	–
Resonance frequency	0.825 (linear)**	0.811 (log)**	0.644 (log)*
The ISQ value	–	0.726 (log)*	n.s.
Peak torque	–	–	0.726 (log)*
Pull‐out force	–	–	–

Abbreviations: ISQ, implant stability quotient; linear, linear fitting; log, logarithmic fitting.

* *p* < .01.

** *p* < .001.

## 4 DISCUSSION

This study is the first to investigate the strength of pedicle screw installation using the Laser RFA method. In the study of the biomechanical test materials, the RF is strongly correlated with the ISQ value (Figure 3C), and it was confirmed that it is possible to perform the same measurement using the existing RFA system (Osstell apparatus). We describe two types of approximation lines (linear and logarithmic) for the correlation study between RF and mechanical test forces (peak torque and POF) (scatter plots in Figure 3A,B). The results demonstrate that both approximations are highly correlated, but the logarithmic approximation shows a slightly higher correlation coefficient than the linear approximation.

Amerini et al.^41^ attempted to evaluate the fixation force using the acoustic moment method in a study using industrial bolts. The results show that the acoustic moment method does not increase at a certain level of torque force. In this study, we adopted a logarithmic fitting to simplify the fitting of the above report. In mechanical engineering, the test of bolt loosening using percussion shows that the region where the mechanical test forces are low and the RF increases rapidly (e.g., the region between 0 and 1 peak torque in Figure 3A) reflects the frictional force between the bolt and the bolt insertion object; the region where the mechanical test forces are high and the RF increase is weak (e.g., the area where the peak torque is 1 or higher in Figure 3A), reflects frictional force and the axial force of the bolt added to the frictional force as it continues to increase.^42^ In other words, the RFA may show a different fixation force from the mechanical method.

In the future use of laser RFA, it will be necessary to analyze the effects of these two forces (frictional force and axial force) on the screw during fixation by combining clinical data/screw mechanics testing and finite element analysis. Nakashima et al.^31^ reported that the ISQ value reflects the resistance against a force in the radial direction of the screw, unlike the mechanical test forces, which reflect axial load.

In the dentistry field, the ISQ value has negatively correlated with the displacement of an implant after the application of a lateral load.^14^, ^16^ This suggests that the ISQ value reflected screw stability against loading in the tangential plane, that is, screw toggle. In vivo, stress forces against the implant were applied in various directions, and not just axial.^43^ Therefore, the mechanical fixation force measuring inserted torque and POF do not necessarily reflect the stress forces in vivo.^43^ Thus, the ISQ value represents a multidirectional fixation force.^17^

RF, such as the ISQ value, was indicated to be a type of index that had different characteristics from those of the mechanical fixation force measures. As described above, peak torque and POF may represent different phenomena from RF and ISQ. However, as a logarithmic fitting was used in this study, it is possible to say that RFA and ISQ are sensitive in the region of low peak torque and POF, while RF is not sensitive in the region of high peak torque and POF. In future, there should be investigations to increase the sensitivity of the RFA, particularly in the region of high peak torque and POF.

In the study using cadaveric vertebrae, the comparison with the ISQ value showed the same high correlation with linear approximation as the studies using the biomechanical (artificial bone model) test materials (Figure 3F). However, there was no significant difference between the two approximation methods compared with the peak torque and POF (Figure 3D,E). When assessing the cadaveric study's test force values, it is possible that only a linear approximation is sufficient because we could obtain only low‐test force values defining frictional force in the biomechanical test materials experiment. Furthermore, although the RF has a constant and sufficient correlation with peak torque and POF, it is more variable than the results obtained with the biomechanical test materials experiments. This may be because peak torque and POF reflect the strength only in the axial direction, whereas the RF reflects the strength in the lateral tilt direction radial to the axis. Thus they may contain bone information from vertebral bones with greater diversity than biomechanical test materials.

Nakashima et al. investigated the correlation between the three test forces of peak torque, POF and ISQ values, and imaging parameters (bone density and bone morphometry with microcomputed tomography (CT)/multidetector CT).^39^ In another study, the characteristics of the three test forces were investigated using a bone model with progressively increased degrees of loosening.^31^ In this loosening model, the degree of interdigitation is gradually reduced by intensifying the loosening. The former study shows that peak torque and POF correlate most strongly with a type of bone morphometry parameter: bone surface/total volume (BS/TV), and the ISQ value correlates with a similar type of parameter: number of nodes (branch points) of the cancellous bone network/total volume (NNd/TV).^39^ The latter study concluded that the ISQ value shows a different method of decreasing with enhanced loosening and by reducing the degree of interdigitation compared to mechanical test forces (insertion torque and POF).^31^ Because laser RFA correlated strongly with the ISQ value in this study, it is expected that laser RFA will behave similarly to the ISQ value. Future detailed bone information in clinical studies and frequency analysis may allow for a more specific assessment of the fixation force.

In addition to insertion torque and POF, there are other methods of measuring implant fixation force, such as the toggle test,^44^ which is a fixation force test for cyclic motion that has different characteristics from the above methods. There are also attempts to predict from imaging parameters, and there are studies that attempt to define fixation force from dual‐energy X‐ray absorptiometry^45^ and CT imaging values.^13^ In addition to the above, the fixation force depends on the design of the screw itself (diameter^46^ and length^47^), the direction of insertion, and the position of placement.^48^ To complicate matters, the required fixation force varies depending on the number of fixed vertebra and the level of vertebrae,^49^ especially for the pedicle screw. As described above, there are various factors that define the fixation force. On the other hand, among these indices for evaluating fixation force, none of these fixation force evaluation indices have yet been used with full consensus from surgeons. The reasons for this may include the complexity, invasiveness, and lack of validity of the measurement methods, which prevents the availability of large amounts of data for research.

Laser RFA has several advantages over previous pedicle screw fixation evaluation systems. First, the laser system is a noncontact system and requires short steps to perform RFA. Laser RFA requires only 10 s from laser irradiation to frequency spectrum analysis. However, the hammer method is more complex as it needs 16 impacts to obtain sufficient data for analysis.^25^ Second, the laser system can be used to irradiate a narrow area repeatedly. The impact force with the hammer is different with each strike,^23^ whereas the laser has the same energy in each irradiation. This is an advantage for obtaining precise data by averaging. The benefits of laser RFA are particularly important when applied to pedicle screw stability during surgeries, which are performed in the limited working space of the human body. These features may be advantageous in the future when large amounts of fixation force data are acquired. Comparisons between laser RFA and the other three test forces are summarized in Table S1.

In dentistry, the ISQ values correlate with the percentage of the implant in contact with the bone (i.e., bone‐to‐implant contact ratio).^50^, ^51^ Using this property, the ISQ value has been used to measure, in vivo, the temporal changes in implant stability that accompany the changes in bone structure around the implant and provide a diagnosis of implant loosening.^14^, ^16^ Therefore, this device will have exploratory use in clinical applications to investigate characteristics exhibited in the pedicle screws and will add value to the investigation of the association of pedicle screw fixation with long‐term implant failure. Finally, it may be possible to set the fixation threshold using laser RFA by obtaining results, such as no screw loosening even in long‐term follow‐ups when the RF is above a certain value.

Two important factors for ensuring final stability have been reported, primarily in dentistry using the ISQ value: initial fixation at the time of implant placement and postoperative osseointegration.^52^ Unfortunately, there are no such studies in the field of spinal surgery, partly because there are no solid intraoperative implant fixation force assessment methods to build upon. In spine surgery as well as in dentistry, two factors can be expected to play a role in the final stability: initial fixation at the time of implant placement and postoperative osseointegration. This device can be the beginning of such implant research.

**Figure 4:**
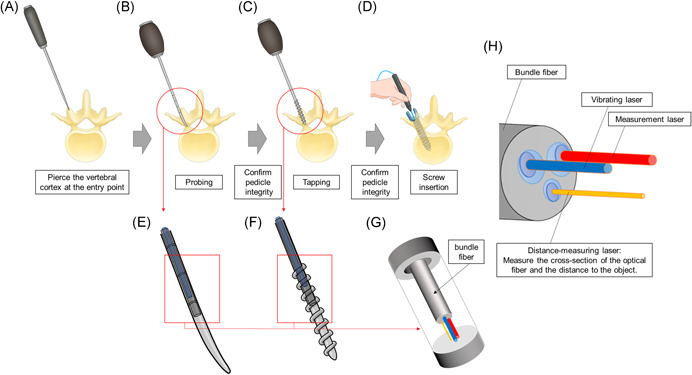
Adaptation of a future resonance frequency analysis system for a series of pedicle screw insertion procedures. A, First, the vertebral cortex is pierced at the entry point with an awl. B, Second, probing is conducted using a pedicle probe. C, After confirming the pedicle integrity by a sounder, tapping is performed. D, After re‐confirming the pedicle integrity, a pedicle screw was inserted. The resonance frequency is then acquired by a pen‐type resonance frequency analysis (RFA) device. E and F, RFA can be implemented not only for pedicle screws but also by incorporating the equipment into probes and taps. G, The laser is guided by a hollow structure of probes and taps with an optical fiber inside (a bundle of optical fibers is placed inside the probe or tap, and the laser emitted from the tip of the bundle fiber reaches the blind end of the hollow structure). H, In this study, we used the vibrating and measurement types of lasers; in the actual system, we used three types of lasers in addition to the vibrating and measurement lasers. [Color figure can be viewed at wileyonlinelibrary.com]

A future RFA system for a series of pedicle screw insertion procedures is shown in Figure 4. Currently, we are considering not only the use of pedicle screws but also the measurement of probing and tapping, which can be used to select the type of screw, determined by the number of fixed vertebrae. This measurement technique determines whether or not to use the S2‐ara‐iliac screw implantation^53^ or the augmentation screw,^43^ which is currently under development. Our RFA system may also make it possible to visualize the soundness of the local bone structure by irradiating the bone itself. If the underlying bone is inadequate for screw insertion due to osteoporosis, another method of stabilization could be used rather than a pedicle screw. The initial clinical significance of laser RFA is that it provides the surgeon with an objective fixation force of the placed pedicle screw. In the next stage, laser RFA can be performed on the tap to determine the actual size (length and diameter) of the pedicle screw to be placed. In addition to the above, the accumulation of clinical data would enable surgeons to predict the prognosis of implant surgery based on intraoperative data, which may lead to new implant treatment strategies.

This study has the following limitations: First, we used cadaveric vertebrae with conditions that differed from those of living bone and were different from clinical conditions. In contrast to the cadaveric bone, in actual surgery, the vertebrae are connected to each other by ligaments and intervertebral discs, and paravertebral muscles are present around them. Therefore, the results may differ from this study. Further, in vivo studies are warranted. Second, the RF was evaluated under dry conditions. In particular, the effect of blood exposure to the implant needs to be considered, but the current assumption is that the effect of blood and other light‐mass materials on vibration is expected to be small. For future high‐precision diagnosis, it is necessary to examine the effects of the wet environment caused by biological fluids and blood. Third, the current laser system involves reflection by fixed mirrors making the irradiation range nonflexible.

Because the current system does not allow the direction of laser irradiation to be changed freely, the pedicle screw needs to be placed in a fixture to make it easier to irradiate with the laser. Therefore, the position of the human body needs to be altered vertically and horizontally to evaluate pedicle screw stability. The free movement of the human body in the operating room is not possible rendering assessment difficult. The use of an optical fiber system to enable the operator to irradiate an arbitrary point can help overcome this problem owing to its flexibility. Fourth, although there exists a cyclic test value as well as torque and POF for implant fixation force, this test was not performed in this study. This test may be close to the multidirectional force reflected by the RF and needs to be investigated in the future.

Fifth, we did not examine the difference in direction and location of laser irradiation. Since the RF obtained by laser RFA is the characteristic vibration of the implant, it can be induced regardless of the direction of excitation. Therefore, it is thought that the RF fluctuations because of the direction and location of irradiation do not occur. In this study, laser irradiation was performed from the side of the screw by raising the root of the screw by 5 mm, but if the entire screw was inserted and irradiated from the longitudinal direction, no change in RF was expected. Further research is warranted to investigate the differences in irradiation location and direction.

The experimental subjects were examined in a uniform and reproducible manner. We believe that this study provides a new perspective on the definition of implant fixation force and presents useful information for implant placement strength research and design developments and improved clinical outcomes. In conclusion, laser RFA has the potential to be a new evaluation index for implant fixation force with excellent reproducibility and usability, replacing the conventional test forces.

## CONFLICT OF INTERESTS

The authors declare that there are no conflict of interests.

## AUTHOR CONTRIBUTIONS

Daisuke Nakashima was in charge of all procedure, analysis, and manuscript write‐up. Katsuhiro Mikami was in charge of laser system construction, data gathering, data analysis, and manuscript write‐up. Shunsuke Kikuchi handled cadaver dissection and bone model preparation. Masaharu Nishikino, Toshiyuki Kitamura, and Noboru Hasegawa performed data analysis. Morio Matsumoto, Masaharu Nishikino, and Takeo Nagura took responsibility for conception.

## Supporting information

Supporting information.

## References

[1] Kwok AWL , Finkelstein JA , Woodside T , Hearn T.C. , Hu R.W. Insertional torque and pull‐out strengths of conical and cylindrical pedicle screws in cadaveric bone. Spine. 1996;21:2429‐2434. 8923627 10.1097/00007632-199611010-00004

[2] Mueller TL , van Lenthe GH , Stauber M , Gratzke C. , Eckstein F. , Müller R. Regional, age and gender differences in architectural measures of bone quality and their correlation to bone mechanical competence in the human radius of an elderly population. Bone. 2009;45:882‐891. 19615477 10.1016/j.bone.2009.06.031

[3] Deyo RA , Gray DT , Kreuter W , Mirza S , Martin BI . United States trends in lumbar fusion surgery for degenerative conditions. Spine. 2005;30:1441‐1445; discussion 1446‐1447. 15959375 10.1097/01.brs.0000166503.37969.8a

[4] Weinstein JN , Lurie JD , Olson PR , Bronner KK , Fisher ES . United States' trends and regional variations in lumbar spine surgery: 1992‐2003. Spine. 2006;31:2707‐2714. 17077740 10.1097/01.brs.0000248132.15231.fe PMC2913862

[5] Bredow J , Boese CK , Werner CML , et al. Predictive validity of preoperative CT scans and the risk of pedicle screw loosening in spinal surgery. Arch Orthop Trauma Surg. 2016;136:1063‐1067. 27312862 10.1007/s00402-016-2487-8

[6] Yagi M , Ames CP , Keefe M , et al. A cost‐effectiveness comparisons of adult spinal deformity surgery in the United States and Japan. Eur Spine J. 2017;27(3):678‐684. 28836012 10.1007/s00586-017-5274-5

[7] Lei W , Wu Z. Biomechanical evaluation of an expansive pedicle screw in calf vertebrae. Eur Spine J. 2006;15:321‐326. 15864667 10.1007/s00586-004-0867-1 PMC3489295

[8] Ab‐Lazid R , Perilli E , Ryan MK , Costi JJ , Reynolds KJ. Does cancellous screw insertion torque depend on bone mineral density and/or microarchitecture? J Biomech. 2014;47:347‐353. 24360200 10.1016/j.jbiomech.2013.11.030

[9] Aparicio C , Lang NP , Rangert B. Validity and clinical significance of biomechanical testing of implant/bone interface. Clin Oral Implants Res. 2006;17(suppl 2):2‐7. 16968377 10.1111/j.1600-0501.2006.01365.x

[10] Meredith N , Alleyne D , Cawley P. Quantitative determination of the stability of the implant‐tissue interface using resonance frequency analysis. Clin Oral Implants Res. 1996;7:261‐267. 9151590 10.1034/j.1600-0501.1996.070308.x

[11] Valderrama P , Oates TW , Jones AA , Simpson J. , Schoolfield J.D. , Cochran D.L. Evaluation of two different resonance frequency devices to detect implant stability: a clinical trial. J Periodontol. 2007;78:262‐272. 17274715 10.1902/jop.2007.060143

[12] Huwiler MA , Pjetursson BE , Bosshardt DD , Salvi GE , Lang NP . Resonance frequency analysis in relation to jawbone characteristics and during early healing of implant installation. Clin Oral Implants Res. 2007;18:275‐280. 17355357 10.1111/j.1600-0501.2007.01336.x

[13] Nakashima D , Ishii K , Nishiwaki Y , et al. Quantitative CT‐based bone strength parameters for the prediction of novel spinal implant stability using resonance frequency analysis: a cadaveric study involving experimental micro‐CT and clinical multislice CT. Eur Radiol Exp. 2019;3:1. 30671863 10.1186/s41747-018-0080-3 PMC6342748

[14] Sennerby L , Meredith N. Implant stability measurements using resonance frequency analysis: biological and biomechanical aspects and clinical implications. Periodontology. 2008;47:51‐66. 10.1111/j.1600-0757.2008.00267.x 18412573

[15] Valderrama P , Oates TW , Jones AA , et al. Evaluation of two different resonance frequency devices to detect implant stability: a clinical trial. J Periodontol. 2007;78:262‐272. 17274715 10.1902/jop.2007.060143

[16] Pagliani L , Sennerby L , Petersson A , Verrocchi D , Volpe S , Andersson P. The relationship between resonance frequency analysis (RFA) and lateral displacement of dental implants: an in vitro study. J Oral Rehabil. 2013;40:221‐227. 23278128 10.1111/joor.12024

[17] Capek L , Simunek A , Slezak R , Dzan L. Influence of the orientation of the Osstell transducer during measurement of dental implant stability using resonance frequency analysis: a numerical approach. Med Eng Phys. 2009;31:764‐769. 19297232 10.1016/j.medengphy.2009.02.003

[18] Saha S , Lakes RS . A non‐invasive technique for detecting stress waves in bone using the piezoelectric effect. IEEE Trans Biomed Eng. 1977;24:508‐512. 914290 10.1109/tbme.1977.326161

[19] Nokes LD . The use of low‐frequency vibration measurement in orthopaedics. Proc Inst Mech Eng. 1999;213:271‐290. 10.1243/0954411991534979 10420780

[20] Georgiou AP , Cunningham JL . Accurate diagnosis of hip prosthesis loosening using a vibrational technique. Clin Biomech. 2001;16:315‐323. 10.1016/s0268-0033(01)00002-x 11358619

[21] Pastrav LC , Jaecques SVN , Jonkers I , Van der Perre G , Mulier M. In vivo evaluation of a vibration analysis technique for the per‐operative monitoring of the fixation of hip prostheses. J Orthop Surg. 2009;4:10. 10.1186/1749-799X-4-10 PMC2678089 19358703

[22] Henys P , Capek L , Fencl J , Prochazka E. Evaluation of acetabular cup initial fixation by using resonance frequency principle. Proc Inst Mech Eng H. 2015;229:3‐8. 25655952 10.1177/0954411914561485

[23] Michel A , Bosc R , Meningaud JP , Hernigou P , Haiat G. Assessing the acetabular cup implant primary stability by impact analyses: a cadaveric study. PLOS One. 2016;11:e0166778. 27893757 10.1371/journal.pone.0166778 PMC5125605

[24] Henys P , Capek L. Impact Force, Polar gap and modal parameters predict acetabular cup fixation: a study on a composite bone. Ann Biomed Eng. 2018;46:590‐604. 29340934 10.1007/s10439-018-1980-3

[25] Tijou A , Rosi G , Hernigou P , Flouzat‐Lachaniette CH , Haïat G. Ex vivo evaluation of cementless acetabular cup stability using impact analyses with a hammer instrumented with strain sensors. Sensors. 2017;18:18. 10.3390/s18010062 PMC5796378 29280982

[26] Lippmann RK . The use of auscultatory percussion for the examination of fractures. JBJS. 1932;14:118‐126.

[27] Leuridan S , Goossens Q , Vander Sloten T , et al. Vibration‐based fixation assessment of tibial knee implants: a combined in vitro and in silico feasibility study. Med Eng Phys. 2017;49:109‐120. 28870418 10.1016/j.medengphy.2017.08.007

[28] Goossens Q , Leuridan S , Henyš P , et al. Development of an acoustic measurement protocol to monitor acetabular implant fixation in cementless total hip arthroplasty: a preliminary study. Med Eng Phys. 2017;49:28‐38. 28760407 10.1016/j.medengphy.2017.07.006

[29] Mikami K , Nakashima D , Kikuchi S , et al. Stability Diagnosis of Orthopedic Implants Based on Resonance Frequency Analysis With Fiber Transmission of Nanosecond Laser Pulse and Acceleration Sensor. Proc SPIE. 2020;11233:112330O-1-6.

[30] Kikuchi S , Mikami K , Nakashima D , et al. Laser resonance frequency analysis: a novel measurement approach to evaluate acetabular cup stability during surgery. Sensors. 2019;19:4876. 10.3390/s19224876 PMC6891423 31717400

[31] Nakashima D , Ishii K , Matsumoto M , Nakamura M. , Nagura T. A study on the use of the Osstell apparatus to evaluate pedicle screw stability: an in‐vitro study using micro‐CT. PLoS One. 2018;13:e0199362. 29953480 10.1371/journal.pone.0199362 PMC6023144

[32] Mikami K , Hasegawa N , Okada H , Kondo S , Nishikino M , Kawachi T. Flash‐lamp‐pumped 4 J, 50 Hz Nd:YAG nanosecond laser system for mobile and transportable equipment. Jpn J Appl Phys. 2017;56:56.

[33] Shimada Y , Kawachi T , Nishikino M , et al. Demonstration of 25‐Hz‐inspection‐speed laser remote sensing for internal concrete defects. J Appl Remote Sens. 2018;12(1):015009‐1–10. https://www.spiedigitallibrary.org/journals/journal-of-applied-remote-sensing/volume-12/issue-01/015009/Demonstration-of-25-Hz-inspection-speed-laser-remote-sensing-for/10.1117/1.JRS.12.015009.full?SSO=1

[34] Katsuhiro M , Noboru H , Toshiyuki K , et al. Characterization of laser‐induced vibration on the concrete surface toward high efficient laser remote sensing. Jpn J Appl Phys. 2020.59 076502‐1–9. https://iopscience.iop.org/article/10.35848/1347-4065/ab9849

[35] Gibson LJ , Ashby MF. Cellular solids: Structure and Properties. 2nd ed. Cambridge, NY: Cambridge University Press; 1997. xviii, 510p.

[36] Paik H , Dmitriev AE , Lehman RA , et al. The biomechanical effect of pedicle screw hubbing on pullout resistance in the thoracic spine. Spine J. 2012;12:417‐424. 22480532 10.1016/j.spinee.2012.03.020

[37] Matsukawa K , Yato Y , Kato T , Imabayashi H , Asazuma T , Nemoto K. In vivo analysis of insertional torque during pedicle screwing using cortical bone trajectory technique. Spine. 2014;39:E240‐E245. 24253778 10.1097/BRS.0000000000000116

[38] Daftari TK , Horton WC , Hutton WC . Correlations between screw hole preparation, torque of insertion, and pullout strength for spinal screws. J Spinal Disord. 1994;7:139‐145. 8003831 10.1097/00002517-199407020-00007

[39] Nakashima D , Ishii K , Nishiwaki Y , et al. Quantitative CT‐based bone strength parameters for the prediction of novel spinal implant stability using resonance frequency analysis: a cadaveric study involving experimental micro‐CT and clinical multislice CT. Eur Radiol Exp. 2019;3:1. 30671863 10.1186/s41747-018-0080-3 PMC6342748

[40] Nelissen RC , Wigren S , Flynn MC , Meijer GJ , Mylanus EAM , Hol MKS . Application and interpretation of resonance frequency analysis in auditory osseointegrated implants: a review of literature and establishment of practical recommendations. Otol Neurotol. 2015;36:1518‐1524. 26375975 10.1097/MAO.0000000000000833

[41] Amerini F , Meo M. Structural health monitoring of bolted joints using linear and nonlinear acoustic/ultrasound methods. Struct Health Monitor. 2011;10:659‐672.

[42] Sah SM , Thomsen JJ , Brøns M , Fidlin A , Tcherniak D. Estimating bolt tightness using transverse natural frequencies. J Sound Vib. 2018;431:137‐149.

[43] Tai C‐L , Tsai T‐T , Lai P‐L , Chen Y.L , Liu MY , Chen LH . A biomechanical comparison of expansive pedicle screws for severe osteoporosis: the effects of screw design and cement augmentation. PLOS One. 2016;10:e0146294. 10.1371/journal.pone.0146294 PMC4697834 26720724

[44] Pelletier MH , Bertollo N , Al‐Khawaja D , Walsh WR . The contribution of the cortical shell to pedicle screw fixation. J Spine Surg. 2017;3:184‐192. 28744499 10.21037/jss.2017.06.07 PMC5506321

[45] Cook S. Biomechanical study of pedicle screw fixation in severely osteoporotic bone. Spine J. 2004;4:402‐408. 15246300 10.1016/j.spinee.2003.11.010

[46] Zhang QH , Tan SH , Chou SM . Investigation of fixation screw pull‐out strength on human spine. J Biomech. 2004;37:479‐485. 14996559 10.1016/j.jbiomech.2003.09.005

[47] Zindrick MR , Wiltse LL , Widell EH , et al. A biomechanical study of intrapeduncular screw fixation in the lumbosacral spine. Clin Orthop Relat Res. 1986;203:99‐112. 3956001

[48] Wu Z , Gao M , Sang H , et al. Surgical treatment of osteoporotic thoracolumbar compressive fractures with open vertebral cement augmentation of expandable pedicle screw fixation: a biomechanical study and a 2‐year follow‐up of 20 patients. J Surg Res. 2012;173:91‐98. 21067776 10.1016/j.jss.2010.09.009

[49] Guo H , Tang Y , Guo D , et al. Pedicle screw fixation in single‐level, double‐level, or multilevel posterior lumbar fusion for osteoporotic spine: a retrospective study with a minimum 2‐year follow‐up. World Neurosurgery. 2020;140:e121‐e128. 32376379 10.1016/j.wneu.2020.04.198

[50] Degidi M , Daprile G , Piattelli A. Determination of primary stability: a comparison of the surgeon's perception and objective measurements. Int J Oral Maxillofac Implants. 2010;25:558‐561. 20556255

[51] Park IP , Kim SK , Lee SJ , Lee JH . The relationship between initial implant stability quotient values and bone‐to‐implant contact ratio in the rabbit tibia. J Adv Prosthodont. 2011;3:76‐80. 21814615 10.4047/jap.2011.3.2.76 PMC3141122

[52] Suzuki S , Kobayashi H , Ogawa T. Implant stability change and osseointegration speed of immediately loaded photofunctionalized implants. Implant Dent. 2013;22:481‐490. 24021973 10.1097/ID.0b013e31829deb62

[53] Chang TL , Sponseller PD , Kebaish KM , Fishman EK . Low profile pelvic fixation: anatomic parameters for sacral alar‐iliac fixation versus traditional iliac fixation. Spine. 2009;34:436‐440. 19247163 10.1097/BRS.0b013e318194128c

