# Supplementary material for: Laser resonance frequency analysis of pedicle screw stability: A cadaveric model bone study
Source: J Orthop Res. 2021 Jan 28;39(11):2474–84. doi: 10.1002/jor.24983 (PMC8596623; doi:10.1002/jor.24983)
Supplement: Supplementary file 1 — Supporting information. [file JOR-39-2474-s001.docx]

Title: Laser resonance frequency analysis of pedicle screw stability: a cadaveric model bone study

Supplementary Materials

Figure S1. Linear and logarithmic correlation analyses among the three fixation force measures (peak torque, pull-out force, and implant stability quotient value)


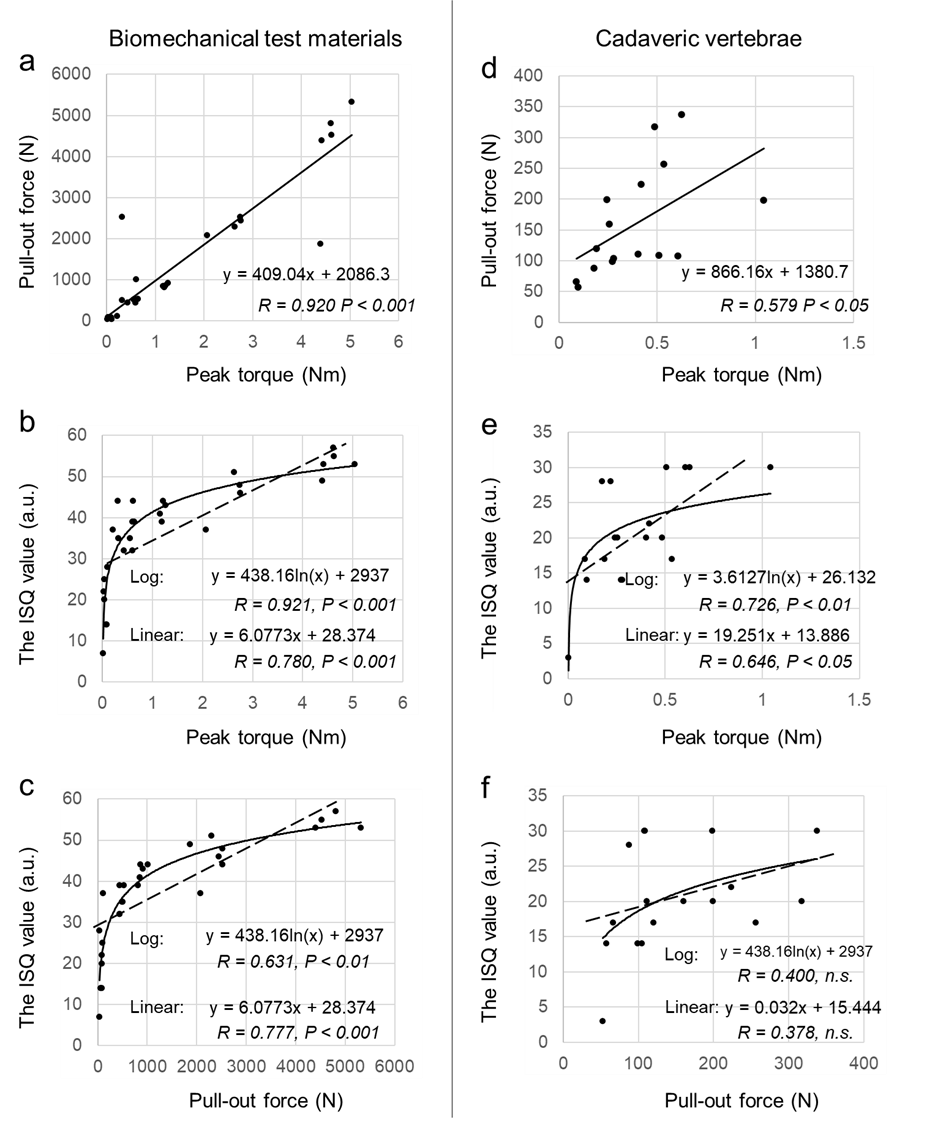


(a and d) Peak torque (Nm) versus pull-out force (N). (b and e) peak torque (Nm) versus implant stability quotient (ISQ) value (a.u.). (c and f) pull-out force (N) versus the ISQ value (a.u.). The top row indicates the study using biomechanical test materials. The bottom row indicates the study using cadaveric vertebrae. N: newton, Nm: newton meter, a.u.: arbitrary units

Table S1. Comparisons of four test forces

|  | Invasiveness | Repeatability | The need for contact with the implant | Complexity |
| --- | --- | --- | --- | --- |
| Pull-out force | invasive | one time only | requiring contact | It is complicated to set up conditions such as angles and how to fix specimens with vices. |
| Peak torque | non-invasive | one time only | requiring contact | It is difficult to standardize the technique because the measurement value changes depending on how far the torque meter is turned. |
| The ISQ value | non-invasive | any number of times | need to attach the magnet to the implant ahead of time | It is necessary to change the magnet to be used depending on the implant. Magnets need to be attached in advance. |
| Resonance frequency | non-invasive | any number of times | completely non-contact measurement | This is a procedure in which the laser is only irradiated onto the implant. |
